# Effect of the Pyro-Gasification Temperature of Wood on the Physical and Mechanical Properties of Biochar-Polymer Biocomposites

**DOI:** 10.3390/ma13061327

**Published:** 2020-03-14

**Authors:** Ramzi Ayadi, Ahmed Koubaa, Flavia Braghiroli, Sébastien Migneault, He Wang, Chedly Bradai

**Affiliations:** 1 Forest Research Institute, University of Quebec in Abitibi-Temiscaming (UQAT), 445 Boul. University, Rouyn-Noranda, QC J9X 5E4, Canada; ramzi.ayadi@uqat.ca (R.A.); sebastien.migneault@uqat.ca (S.M.); he.wang@uqat.ca (H.W.); 2 Technological Center of Industrial Residues (CTRI), Rouyn-Noranda, QC J9X 0E1, Canada; flavia.braghiroli@cegepat.qc.ca; 3 National Engineering School of Sfax (ENIS), University of Sfax, Sfax 3038, Tunisia; chedly.bradai.enis@gmail.com

**Keywords:** biocomposites, pyro-gasification, biochar, physical and mechanical properties, hydrophobicity

## Abstract

The physical and mechanical properties of wood (WPC) and biochar polymer composites (BPC) obtained at different pyro-gasification temperatures and different fiber proportions were investigated. Composite pellets made from wood chips or biochar and thermoplastic polymers (polypropylene or high-density polyethylene) were obtained by twin-screw extrusion, and test specimens were prepared by injection molding. Results showed that BPCs were more dimensionally stable compared to WPCs, but their mechanical properties decreased with increasing pyro-gasification temperatures due to the poor adhesion between the polymer and biochar. Indeed, FTIR investigations revealed the decrease or absence of hydroxyl groups on biochar, which prevents the coupling agent from reacting with the biochar surface. The change in the biochar chemical structure led to an improvement in the dimensional stability and hydrophobicity of the biocomposites. Despite the increased dimensional stability of BPCs compared to WPCs, BPCs still adsorb water. This was explained by the surface roughness and by the biochar agglomerations present in the composite. In conclusion, the thermochemical conversion of black spruce wood chips into biochar makes it brittle but more hydrophobic, thereby reducing the wettability of the BPCs.

## 1. Introduction

Wood-polymer composites (WPCs) are widely used in the automotive and construction fields (e.g., roofing, walls, insulation, flooring), and in the packing and transportation industries [1]. The global WPC market is projected to rise by 13.2% over the next decade to reach approximately $9.7 billion by 2025 [2]. Lignocellulosic materials offer several advantages such as lightness, low density and low cost, whereas thermoplastics generally used in WPCs are melted and processed at temperatures lower than the degradation of lignocellulosic materials (around 200 °C) [3]. Consequently, the combination of wood and plastic provides dimensional stability and strength and contributes to the production of more sustainable materials. The end-use applications of WPCs are greatly related to their mechanical and physical properties such as strength, hardness and impact resistance. Over the past decades, considerable research has been carried out on WPCs with the goal of obtaining good properties, including a low-friction coefficient and abrasion, good plasticity, thermal stability and fire resistance [4]. However, their poor mechanical properties have limited their use to non-structural applications.

Other fillers (wood fibers, glass fibers, calcium carbonate and carbon black) have been investigated to enhance the mechanical properties of composites. For example, filler loading of carbon black can lead to composites with improved mechanical and physical properties as well as cost reduction for the composites [5]. Carbon black, a product of the incomplete combustion of hydrocarbon gases and vapors derived from petroleum, has been one of the most used fillers for decades due to its purity, homogeneity, surface reactivity and low-cost [6]. According to Leblanc [7], the factors that influence its reinforcement capability are: (1) Van der Waal forces between the filler and the polymer, (2) chemical crosslinking between the fiber and the polymer and (3) mechanical interlocking of the polymer to the filler surface. 

Renewable fillers have been suggested in an attempt to lower the use of fossil fuel derived materials in the plastic composite market. Biochar, for example, a carbon-rich material produced from biomass thermochemical conversion processes (i.e., torrefaction, slow to fast pyrolysis, gasification) in a limited oxygen environment, has been used in soil amendments [8], bioenergy (in pellet form) [9] and site restoration [10], as well as a precursor for the production of highly porous activated biochar for water treatment [11,12], energy storage [13], catalysis [14] and electrochemistry [15]. Indeed, to make biochar production profitable in biorefineries or related industries, a multitude of applications should be available. Its advantageous properties, such as hydrophobicity, thermal stability and low to moderate porosity, can have a positive effect on the performance of thermoplastic polymer composites. In addition, the use of biochar in thermoplastic polymer composites could improve the weak properties of WPCs, namely thermal instability, thickness swelling, flammability and poor interfacial adhesion [16].

In a pioneering study, Zaverl et al. [17] prepared plastic composites with wheat straw biochar. Results showed that the addition of 10% biochar slightly increased the tensile and flexural modulus of the composite (~2.7 and ~2.3 GPa, respectively). The use of three types of biochar made from plastic waste (PW), wood shavings (WS) and pine cones (PC) (made using pyrolysis at 450 °C) was investigated for the production of epoxy composites [18]. The highest tensile strength was reported for PW derived materials at a 15% filler concentration (0.59 MPa), and at a 25% concentration for WS and PC epoxy composites (0.51 and 0.64 MPa, respectively). In addition, thermal stability was better at higher filler concentrations (up to 30%). This result suggests that the incorporation of biochar delayed the thermal degradation of the composite and protected the epoxy from heat. 

According to Peterson [19], corn stover biochar based fillers resulted in inferior reinforcement due to the absence of a three dimensional carbon structure, but exhibited more flexibility compared to corn starch based fillers. In a subsequent study, Peterson [20] prepared rubber composites with woody biochar at lower ash contents (2.4%) compared to corn-derived materials, as well as decreased particle size. In this case, biochar-based composites had a better elongation and more flexibility than carbon black filled composites. In addition, higher tensile, elongation and toughness properties of 10% filler concentrations of biochar and carbon black were reached in both studies. Peterson [20] also reported that large particles present in biochar are associated with fracture imperfections and weaknesses in polymer composites. Das et al. [21] noticed that biochar particles were firmly embedded in a PP matrix due to their small particle size (~50 µm).

The studies cited above focused on the introduction of biochar produced from different feedstocks and particle sizes in thermoplastic composites. However, none of these studies focused on different pyro-gasification temperatures during biomass thermochemical modification (i.e., torrefaction or fast pyrolysis) and how this affects biochar physicochemical properties, as well as the physical and mechanical properties of BPCs. Therefore, this study evaluates the effects of torrefied wood residues (315 °C) and biochar (> 315 °C) made from black spruce wood residues on the physical and mechanical properties of BPCs.

## 2. Materials and Methods 

### 2.1. Material Sampling and Biochar Preparation

Polypropylene (PP) Hival 2420 (NexeoSolutions, The Woolands, TX, USA) with a density of 0.903 g cm^−3^, a melting point of 164 °C and a 20 g/10 min melt index, and high-density polyethylene (HDPE) Sclair 2815 (NOVA Chemicals, Calgary, AB, Canada) with a density of 0.952 g cm^−3^, a melting point of 127 °C and a 69 g/10 min melt index, were used for the preparation of the biocomposites. Propylene maleic anhydride copolymer (MAPP) (Admer AT2305A, Mitsui Chemicals America, Rye Brook, NY, USA) with a density of 0.9 g/cm^3^, a melting point of 152 °C and a 1000 g/10 min melt idex at 230 °C, and ethylene maleic anhydride copolymer (MAPE) (DuPont, Wilmington, DE, USA) with a density of 0.93 g cm^−3^, a melting point of 120 °C and a 1.75 g/10 min melt index at 190 °C, were used as coupling agents. Black spruce wood residues were sampled from a sawmill located in Abitibi-Témiscamingue, QC, Canada. Biochar was prepared using CarbonFX technology (Airex Energy Inc., Bécancour, QC, Canada). The torrefaction/fast pyrolysis plant converted wood particles into torrefied wood (315 °C) and biochar (400 and 455 °C) in an oxygen-free environment. More details on biochar preparation have been described elsewhere [22]. 

### 2.2. Preparation of Biocomposites

**Figure 1:**
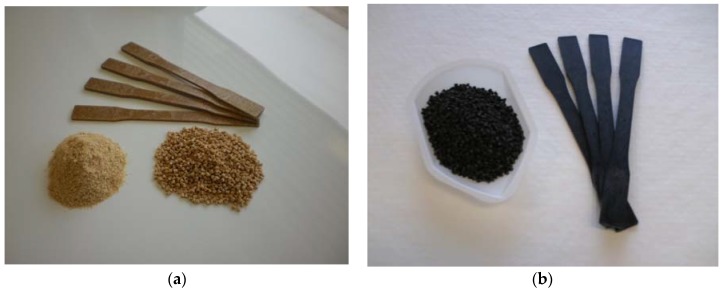
Images of HDPE composite pellets and tensile samples from untreated wood (**a**, wood polymer composites, WPC) and biochar (**b**, Biochar polymer composites, BPC).

Two types of biocomposites (Figure 1) were prepared for comparison purposes: WPCs and BPCs. Wood and biochar particles were first sieved between 100 and 425 μm (40 and 140 mesh). The proportion of both materials varied from 30 to 50%. For example, the loading rate of the various constituents of the composite prepared with 30% filler was: 67% PP with 3% MAPP or 67% HDPE with 3% MAPE, and 30% wood or biochar. Composites were prepared in two stages: compounding for pelletizing followed by injection molding. A counter-rotating intermeshing conical twin-screw extruder (Thermo Scientific HAAKE PolyLab OS Rheodrive 7 with Rheomex OS extruding module, (Thermo Electron GmbH, Karlsruhe, Germany) was used to compound fibers/biochar, PP, HDPE and the coupling agents. The screws were 30 mm in diameter at the large end, and 340 mm long. Screw speed was 30 rpm and the barrel and die temperature was 155 °C. The extruded material was cooled in a water bath and ground into 3 mm long pellets (Figure 1). The pellets were dried for 2 h at 80 °C before conversion into test specimens using conditions illustrated in Table 1. These conditions were based on previous reports [23,24] and dictated by the equipment instructions.

### 2.3. Materials Characterization

Wood particles and biocomposites were analyzed with the Fourier Transformed InfraRed Spectroscopy (FTIR) using a Shimadzu IRTracer˗100 spectrometer equipped with attenuated total reflectance (ATR) (Kyoto, Japon). Elemental determination of carbon, hydrogen, nitrogen and sulfur contents was carried out in a CHNS elemental analyzer (Perkin Elmer 2400 CHNS/O Analyzer; Waltham, MA, USA). Oxygen content was obtained by calculating the difference (%O = 100 − %CHNS). Porosity of black spruce and biochar was obtained by Kr at −196 °C and CO_2_ at 0 °C, respectively, using a Micromeritics ASAP 2460 Surface Area Analyzer (Norcross, GA, USA) after being degassed under vacuum for 48 h at 80 and 100 °C, respectively. Biocomposites fractured surfaces were examined with a HITACHI-S3500N scanning electron microscope (SEM) (Tokyo, Japon). The specimens were first dipped in liquid nitrogen, and then coated with silver prior to the scanning. 

Tensile tests were carried out according to ASTM D638-03 [25] with a test speed of 3 mm min^−1^. Impact tests were carried out in accordance with ASTM D4812-11 [26] using a Zwick/Roell-HIT.5P (Ulm, Germany). Three-point bending tests were performed according to ASTM D790-03 [27]. The distance between the supports was fixed at 80 mm and the load speed at 3.44 mm min^−1^. The equipment used for both tests was a Zwick/Roell Z020 (Ulm, Germany) with a capacity of 20 kN. Water absorption tests of composite specimens were performed according to ASTM D570-98 [28]. Composite specimens were immersed in distilled water at 23 °C for 6 months. Samples were weighed intermittently during this period using an analytical balance (±0.0001 g; Mettler Toledo, Columbus, OH, USA), and the thickness of the specimens was measured with a Starrett micrometer (Athol, MA, USA). Contact-angle measurements were carried out using a Dataphysics OCA 15EC Goniometer (Filderstadt, Germany) with distilled water. The mechanical and physical properties of biocomposites were subjected to variance analysis (ANOVA) with a multivariate linear model. Data were analyzed with SPSS software [29]. Statistical significance was determined using F-tests at *p* ≤ 0.05 and *p* ≤ 0.01.

The surface roughness parameter (Sa, µm) was measured by using a 3D confocal laser scanning microscope (Vk-X150 Kenyence, Itasca, IL, USA.) according to ISO standard 25178. The S-filter (Low-pass filter) was used to eliminate noise or interference present on the original surface. The new surface, called the primary surface, was then treated with the F-operation in Gaussian mode to obtain an S-L surface. Finally, 10 S-L surfaces with a dimension of 1400 µm × 1000 µm were measured for each sample. Equation (1) shows the calculation of *Sa*, where *S* is the S-L surface and *Z(x, y*) is the height obtained at position *x, y*. The porosity (*P*, µm^3^) of all samples was also measured with the help of the same microscope according to the method described by Fredrich [30] and calculated using Equation (2), where *Vvv* represents the void volume of the concave part of the surface and *C.S.*, the cross section (µm^2^).

(1) $$S_{a}=\frac{1}{S}\;\iint_{S}^{\;}\left|z\left(x,y\right)\right|dxdy$$

(2) $$P=\frac{Vvv}{C.S.}$$

## 3. Results

### 3.1. Elemental Composition and Porosity of Black Spruce Wood and Biochar

The physicochemical properties of black spruce and its biochar made at different temperatures are shown in Table 2. It is clear that wood residues undergo a variety of physical, chemical and molecular changes after torrefaction/fast pyrolysis [31]. The carbon content of all materials drastically increased (up 75.4%) compared to wood (48.4%), whereas their oxygen content substantially decreased (up to 19.4 vs. 43.9%). The thermal treatment is also responsible for enhancing the porosity of the biochar through the removal of volatile matter. The highest surface area was reached (208 m^2^ g^−1^) at the highest processing temperature. According to Das et al. [32], the biochar’s porosity may cause pore infiltration by the polymer (e.g., PP), creating mechanical interlocking, and consequently improving mechanical properties of polymer biocomposites. 

**Figure 2:**
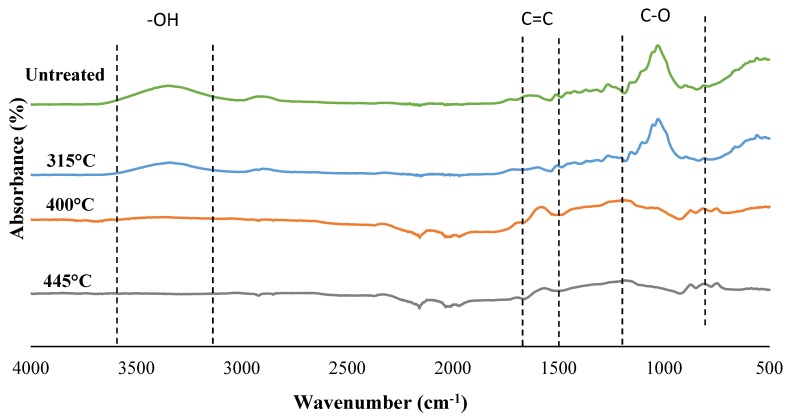
FTIR spectra of spruce wood and biochar treated at 315 °C, 400 °C and 445 °C.

The chemical structure of black spruce and char samples is illustrated by the FTIR spectral data (Figure 2). Firstly, it was observed that wood torrefied at the lowest temperature (315 °C) had almost the same functional groups as wood. For both materials, the first band at 3360 cm^−1^ is attributed to O−H stretching of the hydrogen bonded hydroxyl group [23]. The hydroxyl band decreased drastically in biochar prepared at 400 and 455 °C, which is indicative of their strong hydrophobicity. The band around 1500 and 1600 cm^−1^ is associated with the aromatic C=C skeletal vibration connected to the lignin structure [23]. In biochar spectra, the stretching vibration absorbance of C=C peaks between 1680 and 1580 cm^−1^ may indicate the presence of alkenes [33], whereas the C−O stretching peaks at 1030 and 1160 cm^−1^ are characteristic of C−O−C in cellulose and hemicellulose. The curve shape of biochar prepared at temperatures higher or equal to 400 °C lacked surface functional groups due to the thermal degradation of the wood components (i.e., cellulose, hemicellulose and lignin, partially). 

### 3.2. Mechanical Properties of Biocomposites

**Figure 3:**
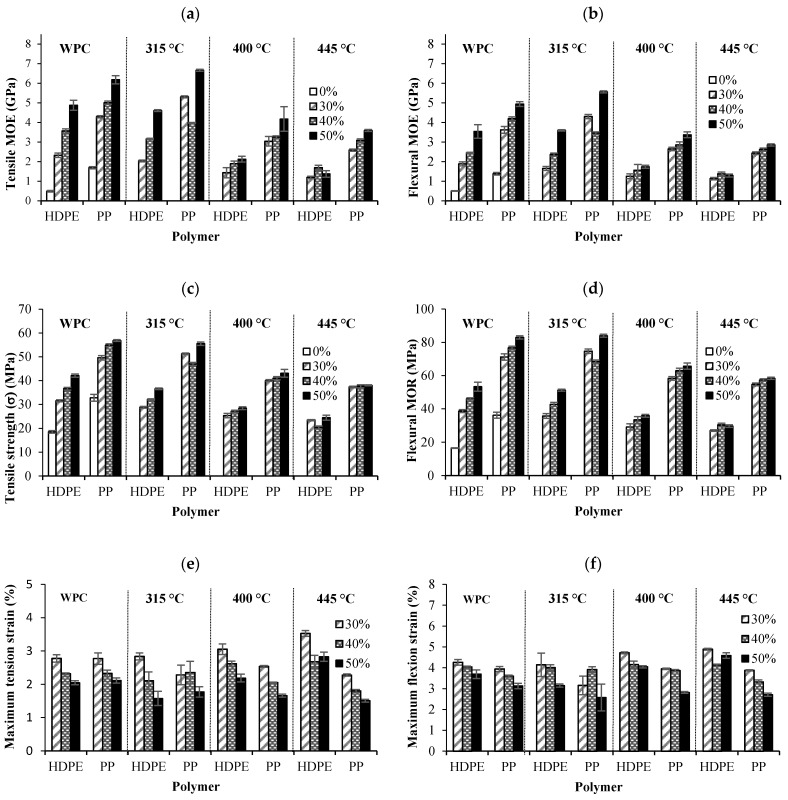

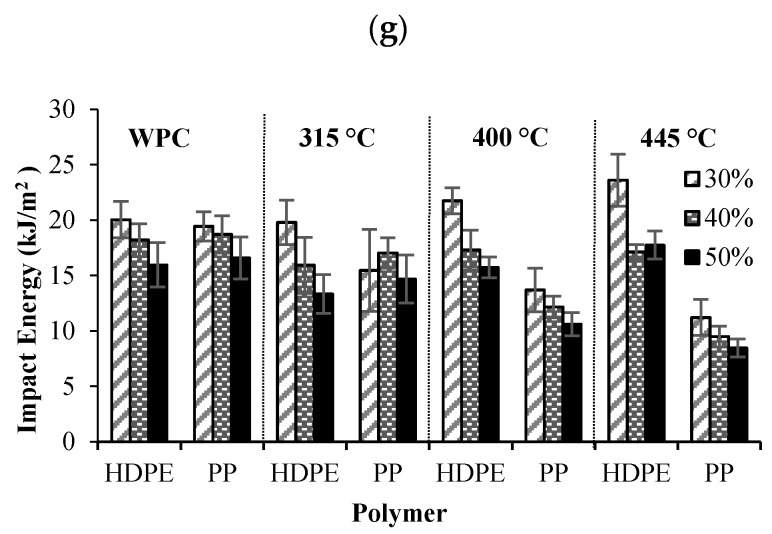
Variation of: (**a**) tensile modulus of elasticity (MOE), (**b**) flexural modulus of elasticity (MOE), (**c**) tensile strength, (**d**) flexural modulus of rupture (MOR), (**e**) maximum tensile strain, (**f**) maximum flexural strain and (**g**) impact energy of biocomposites made with wood and biochar.

Polymer matrix type (PP and HDPE), biochar content (30, 40 and 50%) and pyro-gasification temperature (315, 400 and 454 °C) have significant effects on all mechanical properties (Table 3). The tensile and flexural moduli of elasticity (MOE) and strength are illustrated in Figure 3a,b, and Figure 3c,d, respectively, for WPCs and BPCs. PP biocomposites showed higher mechanical properties than HDPE biocomposites. This was expected since the PP has much higher mechanical properties than HDPE [34]. 

The addition of biochar to PP and HDPE increased the tensile modulus of elasticity and strength (Figure 3a,c). The tensile and flexural strains (Figure 3e,f) were improved with increased pyro-gasification temperature. However, the mechanical properties were enhanced by adding a low percentage of biochar or wood filler (30%) to the HDPE matrix in this case. Higher biochar content decreased the maximum deformation of the biocomposites as well as their impact energy (Figure 3g). At high fast pyrolysis temperatures (≥ 400 °C), the impact energy (Figure 3g) was increased for BPCs in an HDPE matrix, but decreased for BPCs in a PP matrix. 

**Figure 4:**
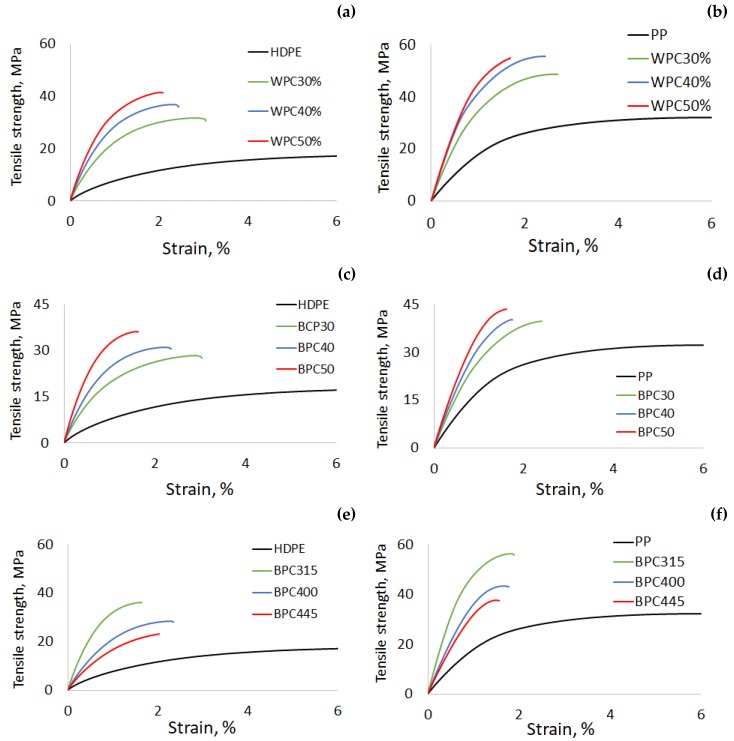
Typical tensile strain-stress curves of wood polymer (WPC) and biochar polymer (BCP) composites: (i) Effect of fiber proportion on (**a**) HPPE and (**b**) PP WPCs, and (**c**) HDPE and (**d**) PP BCPs; and (ii) effect of pyro-gasification temperature on (**e**) HDPE and (**f**) PP BPCs.

**Figure 5:**
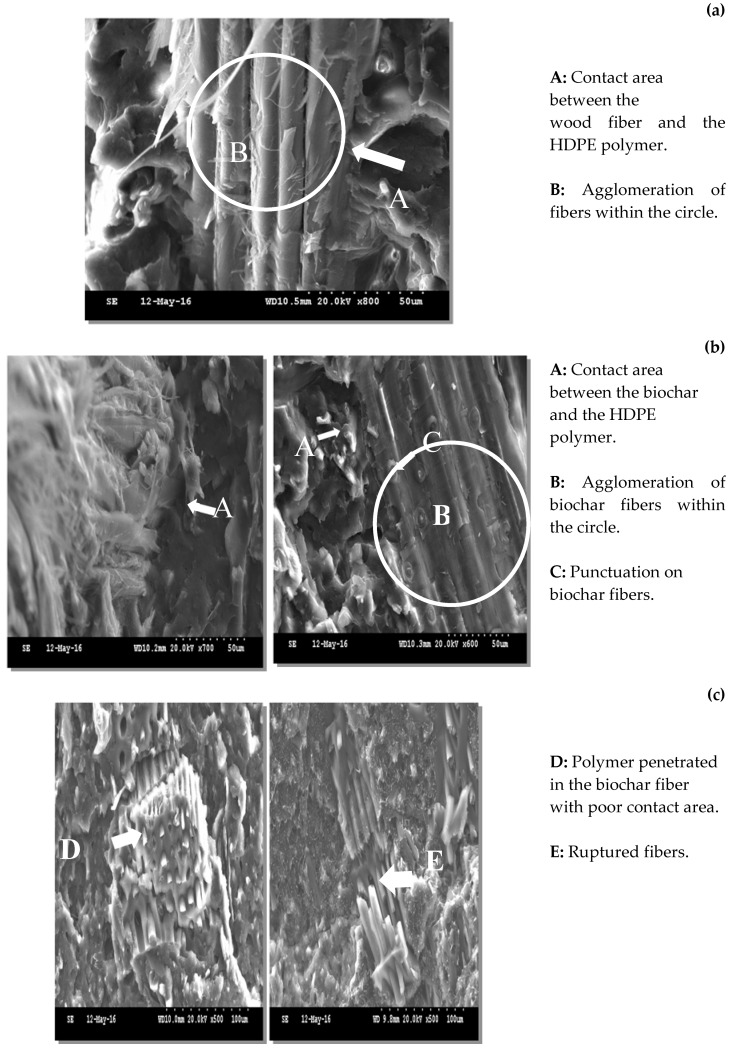
Scanning electron microscope (SEM) images of various HDPE biocomposites: (**a**) WPC with 40% untreated wood fibers; (**b**) BCP with 40% biochar treated at 315 °C; and (**c**) BCP with 40% biochar treated at 400 °C (Left) and 445 °C (Right).

This decrease in impact energy with the addition of wood (Figure 4a,b) and biochar fibers (Figure 4c,d) is related to the low plastic deformation of the biocomposites and weak adhesion to the matrix [35]. SEM observations supports this affirmation (Figure 5a,b). The impact energy of BPCs was lower than that of WPCs because of their lower rigidity and interfacial adhesion. The absence of an interaction between the biochar and coupling agent (MAPP or MAPE) was because of the drastic reduction in oxygenated functional groups connected to the biochar [36]. The disappearance of the peaks associated with hydroxyl groups in the biochar (Figure 2) supports this affirmation.

The mechanical properties decreased with increasing pyro-gasification temperatures. BPCs from biochar prepared at 315 °C showed better or equal tensile and flexural properties compared to WPCs. These values were enhanced by the addition of 50% biochar filler in a PP matrix. At higher temperatures (400 and 445 °C), the impact of biochar proportion on BCPs tensile and flexural properties is marginal compared to that on WPC and BCPs prepared from 315 °C biochar. The better strength properties of the biocomposites made with biochar treated at a lower pyro-gasification temperature (315 °C) could be explained by the presence of hydroxyl groups at the surface of fibers treated at this temperature (Figure 2), which promotes the interaction between the biochar and the coupling agents. The low impact energy of BPCs compared to polymers and WPC (Figure 3g) is due to their lower strength and low plastic deformation (Figure 4e,f). 

### 3.3. Morphology of Biocomposites

SEM images illustrate the effects of torrefaction or fast pyrolysis on the interface between the biochar and the polymer matrix. Firstly, the contact between biochar and the polymer (HPDE) is shown by letters A and B in Figure 5a. The fibers were well embedded in the polymer matrix, likely due to the effect of the coupling agent (MAPP) used in the preparation of the WPC. The thermal treatment of wood at 315 °C (Figure 5b) also showed a close contact between the torrefied wood and the polymer matrix. In this case, the fibers retained some characteristics of the conventional wood structure, such as punctuations (letter C). However, fast pyrolysis at 400 and 445 °C (Figure 5c) completely changed the structure of the wood cells. The polymer penetrated into the biochar particles (letter D), but they became very fragile (letter E). A fracture was identified in both materials, the BPCs made with biochar at 400 and 445°C, letter D (biochar-polymer interface) and letter E (biochar particles), respectively. Similar findings were reported for rice husk biochar thermoplastic composites with 60% biochar content: the interaction between biochar and HDPE became poor and the interface presented some gaps [37]. Therefore, the weak adhesion between biochar (at high proportions or high pyro-gasification temperatures) and the polymer matrix also explains the decrease in mechanical properties of BPCs from the high pyro-gasification treatment. This poor adhesion is explained by the absence of hydroxyl groups on the biochar surface, which are the main functional groups that react with the coupling agent.

### 3.4. Surface Roughness and Porosity

**Figure 6:**
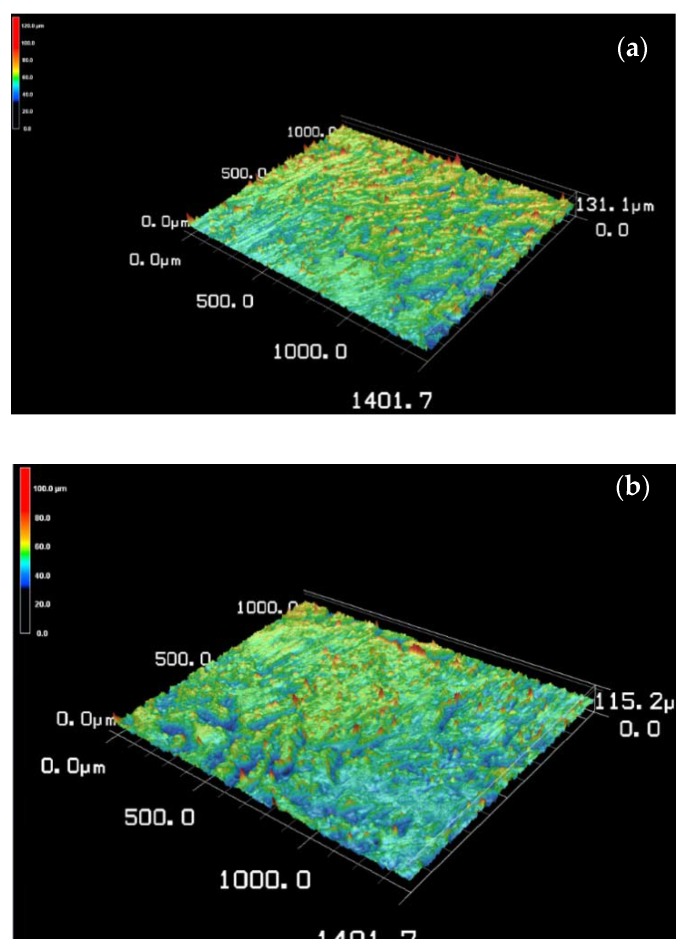

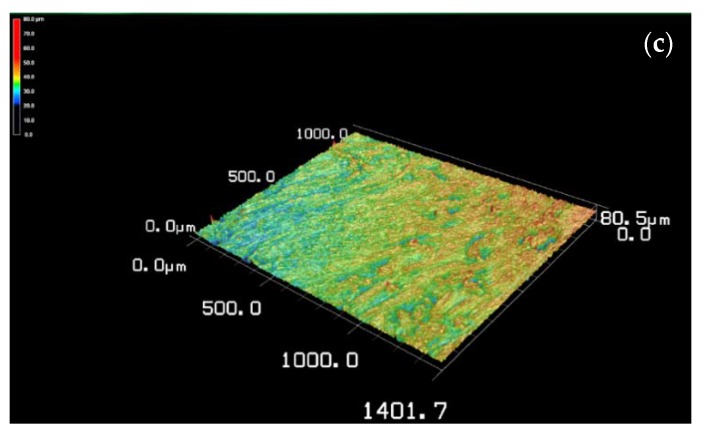
3D laser images of the surface roughness of BPCs made with 30% biochar prepared at 315 °C (**a**), 400 °C (**b**) and 445 °C (**c**).

**Figure 7:**
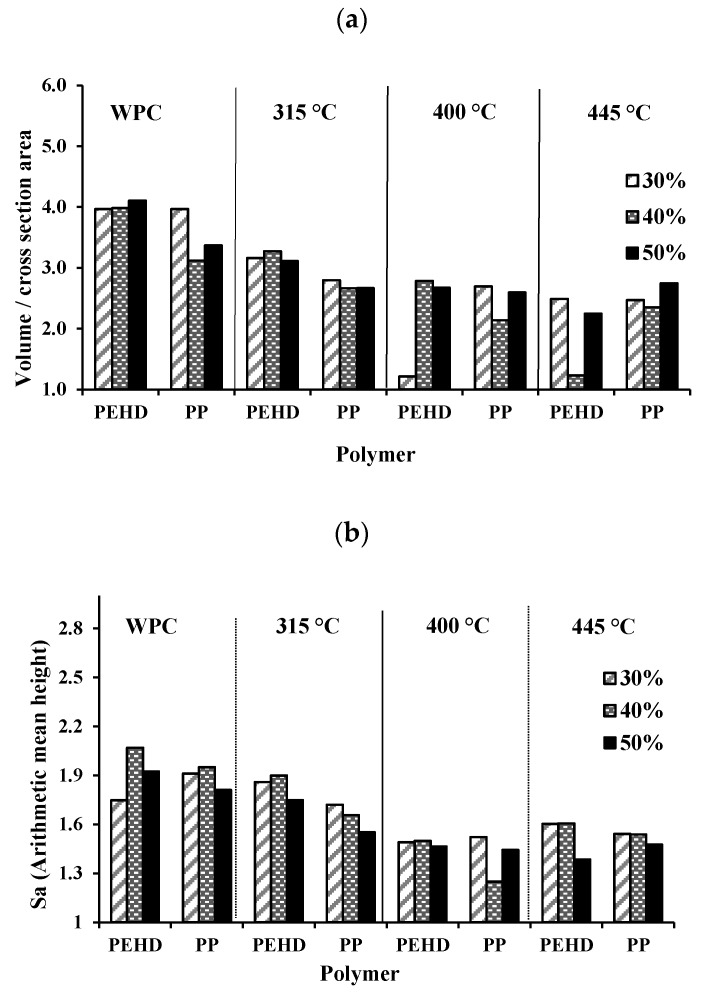

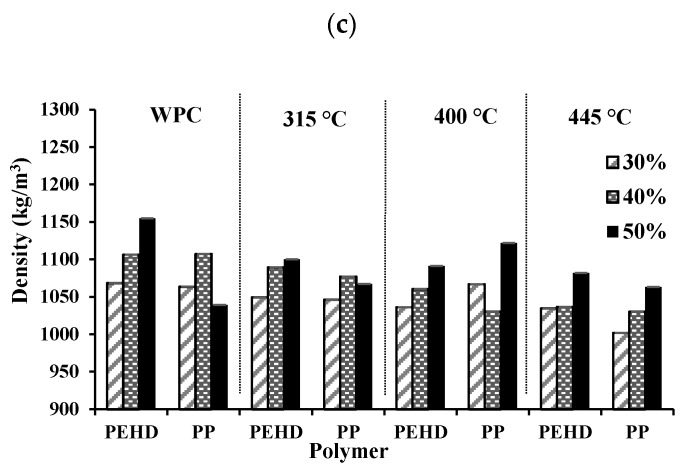
Variation in: (**a**) porosity (Volume/cross section area; µm), (**b**) surface roughness (Sa: Arithmetic mean height; µm) and (**c**) density (kg/m^3^) of biocomposites made with wood and biochar at different temperatures and in different proportions.

A 3D laser confocal microscope was used to measure the surface roughness and porosity of the biochar biocomposites. When comparing the surface roughness obtained for BPCs made using biochar at 315, 400 and 445 °C (Figure 6a–c, respectively), a reduction in peaks higher than 100 µm of height was observed (Figure 6a–c). This means that the dimension of biochar particles was reduced at 445 °C, and this led to a BPC surface that is flatter than at 315 °C. There is also the presence of wood fibers that were not completely transformed into biochar at 315 °C or 400 °C. The advantage of this mixture of biochar and wood fibers is that the surface polarity of BPCs may be increased, thereby improving adhesion with other materials. No wood fibers were identified at 445 °C, which means that all wood fibers were transformed into biochar, making the surface of the BPC flatter and with a reduced waviness as well. The results obtained for porosity (*P*) and surface roughness (*Sa*) for all tested biocomposites are shown in Figure 7a,b, respectively. No obvious trend is observed from the porosity data (Figure 7a) except that the BCPs showed slightly lower porosity values compared to WPCs. The slightly lower porosity values of the BCPs could be explained by the penetration of the polymer in the biochar structure (Figure 5c). The bad dispersion of the biochar in the composites is among the plausible explanations for the absence of trend in porosity data. Further investigations are needed to explain this result.

Table 4 presents the surface roughness classification of the materials. According to this classification, the surface roughness of all BPCs was found to be between 1.0 and 2.0 μm, which is considered rough. Measured *Sa* and *P* decreased with the addition of biochar made at the highest temperature (445 °C) and at the highest proportion (50%). Composite density (Figure 7c) was correlated to both properties. In general, there was a reduction in the density of biocomposites with the addition of wood and biochar compared to neat polymers due to the voids in the wood structures and in the interface wood-polymer.

### 3.5. Water Uptake and Contact Angle

**Figure 8:**
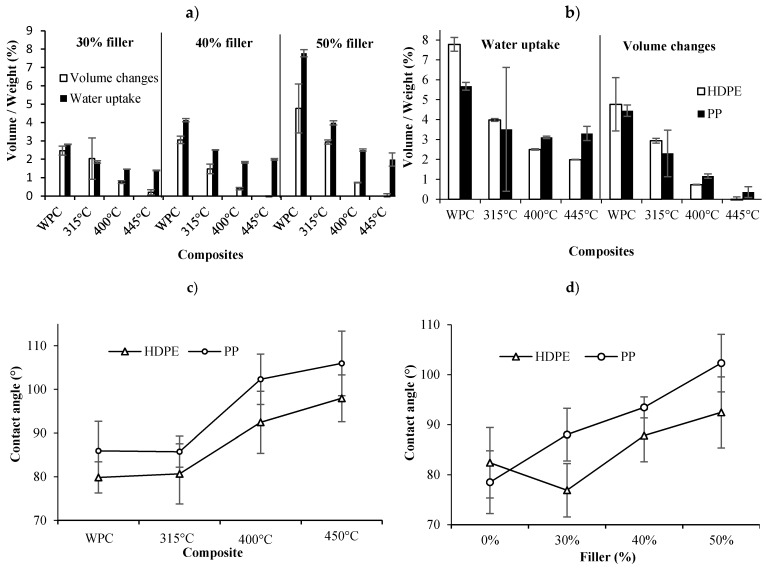
**(a**) Water uptake and volume changes after 6 months immersion water testing with wood and biochar composites made with HDPE; (**b**) effect of polymer matrix (HDPE or PP) on water absorption and volume changes of wood and biochar composites made with 50% filler after 6 months testing; (**c**) effect of pyro-gasification temperature on the contact angle of the same materials in (**b**); (**d**) effect of filler content of BS400PC on the contact angle.

The results of water uptake tests show that water absorption and volume change of WPCs decreased with the addition of biochar (Figure 8a–c). In good agreement with previous findings [24,39], higher fiber proportions for both types of wood biochar fibers led to higher water uptake and volume change. However, biocomposites made with wood particles swelled to a greater extent than those with biochar at all fiber proportions. In addition, BPC samples treated at 450 °C were not dimensionally affected after 6 months of immersion in water (Figure 8b). The reason behind the unchanged volume and low water uptake values for BPCs is that biochar becomes very hydrophobic, due to its physical, chemical and molecular changes, with increasing pyro-gasification temperature [31]. FTIR results (Figure 1) support this affirmation since the hydrophilic OH peaks disappeared in the pyro-gasified fibers. On the other hand, BPC filled biocomposites still absorbed water, but to a lower extent than WPCs. Water settled in the composite voids and gaps, but the biochar fiber walls were hydrophobic and did not absorb water. 

**Figure 9:**
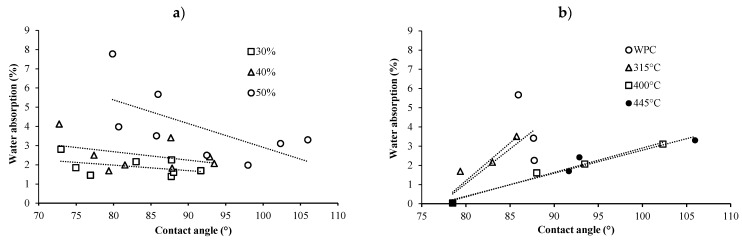
**(a**) Relationship between water uptake and contact angle for composites made at different filler content (30−50%), and (**b**) relationship between water uptake and contact angle for biochar composites made at different pyrolysis temperature (315−445 °C).

Contact angles increased for BPCs prepared with biochar made using fast pyrolysis at temperatures equal to or higher than 400 °C (Figure 8c), as well as at high biochar contents (Figure 8d). The decrease of the contact angle from 0% to 30% biochar content is not statistically significant as indicated by the high standard error and could be explained by experimental errors. However, BPCs did absorb water and, therefore, did not become more hydrophobic with an increase in biochar content (Figure 9a). It has been stated that an increase in filler content may increase surface roughness [40] and, therefore, wettability. However, according to the surface roughness results described previously, the surface roughness of BPCs was considered rough and an increase in filler content reduced surface roughness. In this case, we could assume that there was another factor affecting the water absorption results; it is possible that agglomerations of biochar were present in the final composite that absorbed more water than a homogenous BPC. The relationship between contact angle and water uptake at different biochar contents and pyro-gasification temperatures is illustrated in Figure 9a,b, respectively. The hygroscopicity of biocomposites decreased with an increase in the contact angle at a fixed biochar contents (e.g., 50%), whereas the hygroscopicity of biocomposites increased with an increase in the contact angle when the pyrolysis temperature (e.g., 445 °C) was fixed. Based on these findings, it is concluded that the addition of biochar in BPCs provokes an increase in water absorption and contact angle due to not only surface roughness, but also to biochar agglomerations in the composite, as explained previously.

### 3.6. Practical Implications

Pyro-gasification temperatures related to the CarbonFX technology generate gases, oil and solid residues in the form of torrefied biomass or biochar. In this study, the variation of temperature induced important chemical and surface chemistry changes in wood residues, but had little impact on their porosity. However, these changes had a great influence on the physical and mechanical properties of composites. Specifically, materials had better mechanical properties when prepared at a low temperature (315 °C) compared to 450 °C. This was explained by the chemical composition of torrefied wood and its great proportion of oxygenated groups on its surface that played a crucial role on its interactions with the polymer. Thus, composites made with wood treated at this temperature (315 °C) will be suited for the same applications as those of conventional wood polymer composites made with untreated fibers, but with the advantage of presenting better dimensional stability. 

Although composites made with biochar showed lower mechanical properties, their great dimensional stability is an asset for applications where dimensional changes are not tolerated, such as for door and window skins. For these applications, mechanical properties such as bending and tensile are not critical. Thus, biochar obtained at high pyrolysis temperature (450 °C) would be suited to produce composites for such applications.

## 4. Conclusions

Biochar content (30, 40 and 50%), polymer matrix type (PP and HPDE) and pyro-gasification temperature (315, 400 and 454 °C) had significant effects on the mechanical and physical properties of biochar biocomposites. BPCs were more dimensionally stable compared to those from untreated wood. The significant decrease in mechanical properties observed in BPCs made from fast pyrolysis at temperatures higher than 400 °C is related to differences in their chemical composition and surface chemistry compared to the cell wall structure of wood particles. Chemical modifications of materials were identified by FTIR analyses, where the heat treatment resulted in the disappearance of hydroxyl groups and an increase in aliphatic groups. Consequently, the biochar chemical structure led to an improvement in the dimensional stability and hydrophobicity of the biocomposites. However, the mechanical properties of BPCs decreased with increasing pyro-gasification temperatures. The addition of wood to BPCs, the reduction of biochar particle size and the modification of the biochar chemical and porous structure with chemical treatment and activation are currently being studied to improve such properties.

## Figures and Tables

**Table 1:** Injection molding machine parameters.

Parameters	HDPE	PP
Mold temperature (°C)	20	20
Injection pressure (bar)	900−1325	1300−1400
Hold pressure: (bar)	450−600	600−925
Injection pressure time (s)	0.8−1.9	0.8−1.9
Hold pressure time (s)	11	11
Cooling time (s)	17	17
Total cycle time (s)	33.2−33.6	33.3−34.4
Dosing volume (cm^3^)	18/26/34/41	18.5/26/34/41
Decompression volume (cm^3^)	5	5
Screw speed (RPM)	215	215
Barrel temperature profile–feed, zone1, zone2, nozzle (°C)	170/190/190/190	185/205/205/205

**Table 2:** Physicochemical properties of wood residues and biochar.

	*C* *(%)*	*H* *(%)*	*N* *(%)*	*S* *(%)*	*O* *(%)*	*Specific Surface Area (m^2^ g^−1^)*
Black spruce	48.4	6.57	0.08	1.04	43.9	0.5
315 °C	53.0	5.76	0.74	0.86	39.6	42
400 °C	72.5	3.71	0.71	0.60	23.0	158
445 °C	75.4	3.84	0.87	0.51	19.4	208

**Table 3:** Results of the analysis of variance (F-value) for mechanical and physical properties of biochar biocomposites.

	Tensile			Impact	Three-Point Bending			Contact Angle	Water Uptake	
	MOE	MOR			MOE	MOR			Volume Changes	Absorp-tion
Biochar content (A)	1985 **	4447 **	596 **	1422 **	1009 **	1833 **	120 **	6.9 **	91 **	354 **
Polymer type (B)	2396 **	11759 **	35 **	93 **	1802 **	6042 **	203 *	13 **	0.3 ^ns^	0.2 ^ns^
Temperature (C)	1358 **	90 **	4 *	16 **	688 **	813 **	18 **	20 **	179 **	155 **
A x B	97 **	434 **	1.5 ^ns^	6 **	76 **	215 **	16 **	4 *	1.7 ^ns^	4 **
A x C	104 **	102 **	0.9 ^ns^	1 ^ns^	62 **	33 **	10 **	3 *	9 **	27 **
B x C	13 **	120 **	18 **	32 **	14 **	10 **	14 **	0.9 ^ns^	1.9^ns^	21 **

*: significant at 0.05; **: significant at 0.01; ns: non-significant.

**Table 4:** Classification of materials surface roughness [38].

Surface Roughness value, *Sa* μm	Surface Classification
0–0.4	Smooth
0.5–1.0	Moderately rough
1.0–2.0	Rough
>2.0	Extremely rough
